# Anticancer Mechanisms of Salinomycin in Breast Cancer and Its Clinical Applications

**DOI:** 10.3389/fonc.2021.654428

**Published:** 2021-07-26

**Authors:** Hui Wang, Hongyi Zhang, Yihao Zhu, Zhonghang Wu, Chunhong Cui, Fengfeng Cai

**Affiliations:** ^1^ Laboratory of Tumor Molecular Biology, School of Basic Medical Sciences, Shanghai University of Medicine and Health Sciences, Shanghai, China; ^2^ Department of Breast Surgery, Yangpu Hospital, School of Medicine, Tongji University, Shanghai, China; ^3^ Department of Scientific Research, Shanghai University of Medicine and Health Sciences Affiliated Zhoupu Hospital, Shanghai, China; ^4^ Shanghai Key Laboratory of Molecular Imaging, Shanghai University of Medicine and Health Sciences, Shanghai, China

**Keywords:** salinomycin, breast cancer, mechanism, clinical application, cancer stem cells

## Abstract

Breast cancer (BC) is the most frequent cancer among women worldwide and is the leading cause of cancer-related deaths in women. Cancer cells with stem cell-like features and tumor-initiating potential contribute to drug resistance, tumor recurrence, and metastasis. To achieve better clinical outcomes, it is crucial to eradicate both bulk BC cells and breast cancer stem cells (BCSCs). Salinomycin, a monocarboxylic polyether antibiotic isolated from *Streptomyces albus*, can precisely kill cancer stem cells (CSCs), particularly BCSCs, by various mechanisms, including apoptosis, autophagy, and necrosis. There is increasing evidence that salinomycin can inhibit cell proliferation, invasion, and migration in BC and reverse the immune-inhibitory microenvironment to prevent tumor growth and metastasis. Therefore, salinomycin is a promising therapeutic drug for BC. In this review, we summarize established mechanisms by which salinomycin protects against BC and discuss its future clinical applications.

## Introduction

According to the International Agency for Research on Cancer of the World Health Organization, the number of new breast cancer (BC) cases increased to 2.26 million in 2020, exceeding the number of lung cancer cases (2.21 million in 2020) for the first time; thus, BC has officially replaced lung cancer as the most common cancer worldwide. BC accounts for 1/4 of female cancer cases and 1/6 of female cancer-related deaths. In most countries, it is now the greatest threat to the health of women.

Owing to the limitations of current therapies, many patients die from metastasis, recurrence, and drug resistance (1, 2). In the last two decades, studies have revealed that breast cancer stem cells (BCSCs), a small subpopulation of cells, have been shown to have the ability to self-renew and differentiate into tumor cells. The clinical relevance of BCSCs has been a focus of many studies (3, 4), which have demonstrated that these cells are resistant to conventional chemotherapy and radiation treatment and are very likely to be the origin of cancer relapse and metastasis (5–9). Hence, to improve clinical outcomes, it is crucial to eradicate both bulk BC cells and BCSCs.

**Figure 1:**
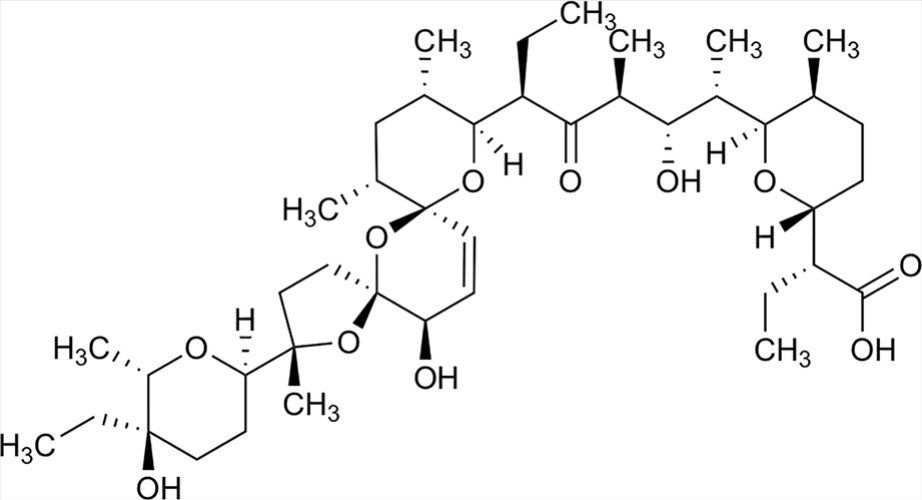
Chemical structure of Salinomycin, edit from (11).

Salinomycin is a monocarboxylic polyether antibiotic derived from *Streptomyces albus* (10). Its chemical structure is shown in **Figure 1**. It functions on different biological membranes and shows high affinity for positive ions, especially potassium, interfering with the balance of ion concentrations between the inside and outside of cells, thereby affecting osmotic pressure and eventually leading to germ cell disruption (12). At the end of the last decade, the preferential toxicity towards cancer stem cells (CSCs) has been reported by Gupta and colleagues (13). In addition to BC, salinomycin can also selectively kill CSCs in many other types of cancers, such as ovarian, lung, prostate, and colorectal cancers (14–19). The multiple function of salinomycin against CSCs and its molecular mechanism have been intensively studied in many types of cancer cells, with these studies extensively reviewed in several publications (11, 20–22). These reviews have also described the chemical properties of salinomycin in detail and summarized its role in cancer cells and CSCs. Extensive studies on BC have established that salinomycin inhibits cell proliferation, invasion, and migration, modulates cell death, and reverses the immune-inhibitory microenvironment to prevent tumor growth and metastasis (13, 16, 23–26). Moreover, salinomycin can be used to kill BC cells with drug resistance (27, 28). Therefore, salinomycin is expected to be a potent therapeutic drug for BC.

In this review, we summarize our current understanding of the mechanisms by which salinomycin inhibits BC, including its precise effects on BCSC proliferation, invasion, migration, and apoptosis. Furthermore, we discuss its potential applications in BC therapy, including the potential utility of nanocarrier-based delivery systems and combination therapies.

## Effects of Salinomycin on BCSCs

CSCs are tumor cells with stem cell characteristics and initiate cancer formation. They form and maintain heterogeneous tumor entities through self-renewal and asymmetric division (29). Compared with highly differentiated tumor cells, CSCs have a lower degree of differentiation, stronger drug metabolism, and the ability to repair drug-induced damage (30). They can renew themselves through epigenetic changes, acquire new mutations, and quickly adapt to the environment, thereby forming a new tumor entity with molecular characteristics very differently from those of the initial tumor. Therefore, CSCs are also considered to be the source of tumor metastasis and drug resistance (31). If therapies only target differentiated tumor cells and cannot effectively eliminate CSCs, tumor recurrence will be inevitable. Hence, effective inhibition of differentiated tumor cells and simultaneous and specific elimination of CSCs are the focus of current research in the field of tumor therapy.

Salinomycin is over 100 times more effective against BCSCs than paclitaxel, the traditional chemotherapy drug for the treatment of BC (26). Salinomycin increases the apoptosis of BC mammosphere cells, which is accompanied by downregulation of Bcl-2 expression, and decreases their migration capacity, which is accompanied by downregulation of c-Myc and Snail expression (23). Furthermore, extensive studies on BCSCs have established that salinomycin decreases CSC population (32–34). Global gene expression analyses have shown that salinomycin inhibits the expression of BCSC genes (13). In addition, consistent with previous findings in prostate cancer, salinomycin reduces aldehyde dehydrogenase activity and the expression of MYC, AR, and ERG; it induces oxidative stress and inhibits nuclear factor (NF)-κB activity (35). In BC cells, salinomycin influences stem cell signaling, such as Wnt and Hedgehog signaling, or ALDH1 activity, to suppress mammosphere formation, induce cell apoptosis, or inhibit cell proliferation (34, 36–39). Further, salinomycin effectively inhibits mammary tumor growth *in vivo*. In BC xenograft tumors, it significantly reduces tumor growth, which is accompanied by decreased PTCH, SMO, Gli1, and Gli2 expression (23).

**Figure 2:**
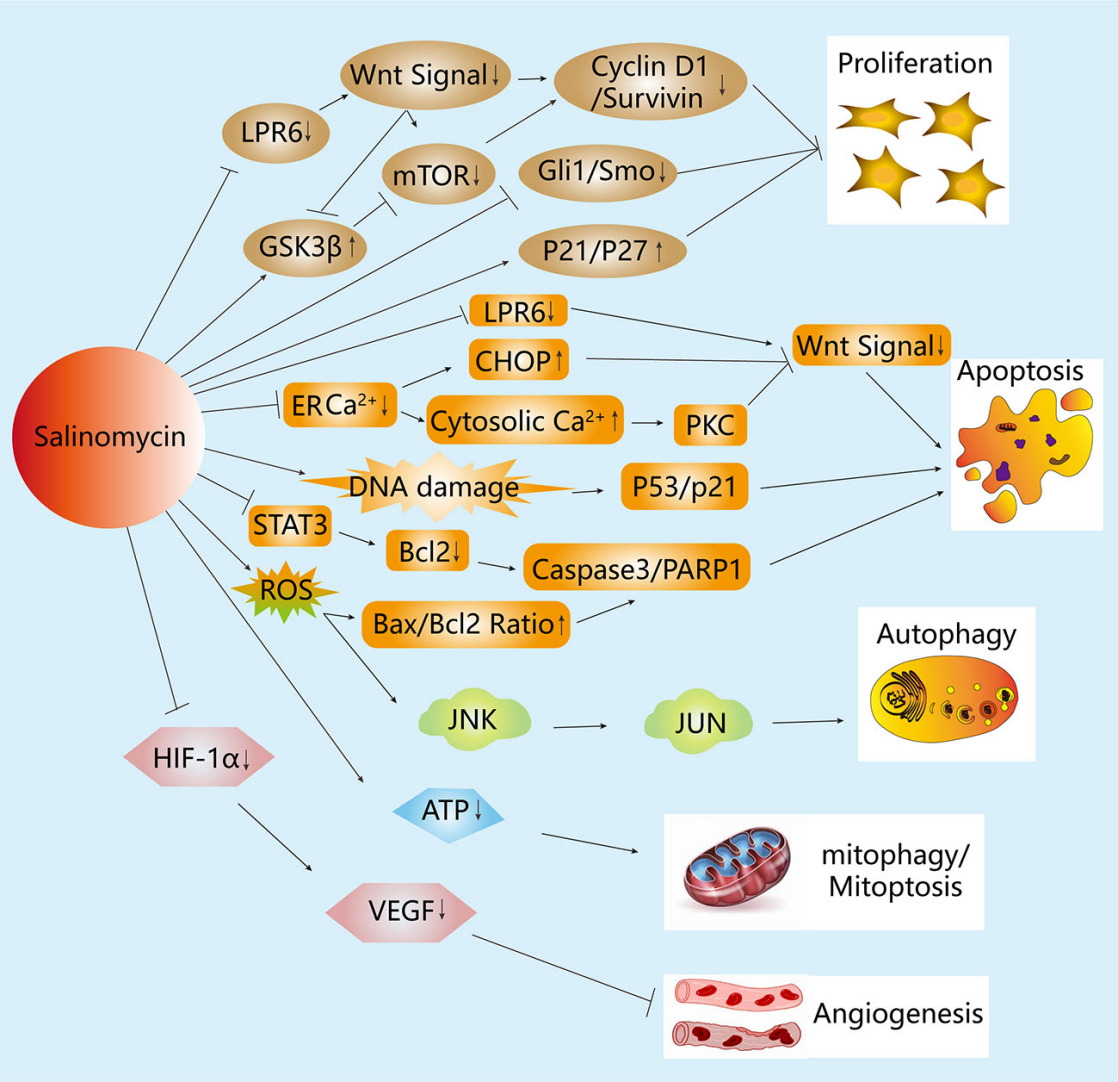
Mechanisms of the anticancer activity of salinomycin in breast cancer (BC) cells. Salinomycin inhibits cell proliferation in BC cells through the Hedgehog and Wnt signaling pathways. Additionally, salinomycin is capable of inhibiting Wnt signaling by blocking the phosphorylation of LRP6 or by ER stress, which leads to apoptosis in BC cells. Moreover, salinomycin induces the apoptosis of BC cells by increasing DNA damage or intracellular ROS level, thereby influencing the p53 and caspase cascade separately. Salinomycin also induces autophagy by increasing intracellular ROS level, which is accompanied by MAPK signaling pathway activation. Furthermore, salinomycin can affect the cell membrane potential and reduce the level of ATP to induce mitophagy and mitoptosis. Moreover, salinomycin inhibits angiogenesis through the VEGF signaling pathway.

Although the exact mechanisms underlying the effects of salinomycin on BCSCs are not fully understood, recent studies have provided insights into its molecular mechanisms and modes of action. The effect of salinomycin on BCSCs and BC cells through various mechanisms of action and its molecular targets have been summarized in this review (see **Tables 1**, **2** and **Figure 2**).

**Table 1:** Anticancer mechanisms of salinomycin in BCSCs.

Mechanisms	BCSCs	Molecular targets of salinomycin	Activity *in vitro/in vivo*	Ref.
Affect iron homeostasis	HMLER CD44^high/^CD24^low^ cells	Salinomycin accumulates and sequesters iron in lysosomes and triggers ferritin degradation in response to iron depletion, further activating a cell death pathway consistent with ferroptosis.	*in vitro* and *in vivo*	(40)
Affect apoptosis, cell growth and migration	MCF7 mammosphere cells	Salinomycin increases apoptosis by a decreased expression of Bcl-2; decreases the migration capacity accompanied by a decreased expression of c-Myc and Snail in MCF7 MS cells, and significantly reduces the tumor growth accompanied by decreased expression of the critical components of the Hedgehog pathway (PTCH, SMO, Gli1 and Gli2) in xenograft tumors.	*in vitro* and *in vivo*	(23)
Affect autophagic flux	ALDH(+) HMLER cells	Salinomycin has a relatively greater suppressant effect on autophagic flux, which may be affected by ATG7 and correlates with an increase in apoptosis.	*in vitro*	(41)
	CD44+/CD24^low^ HMLER cells	Acidic conditions improve the ability of salinomycin to inhibit the autophagic flux and kill CSCs.	*in vitro*	(32)
Induce cell differentiation	MDA-MB-435 mammosphere cells	Salinomycin blocks the PKCa signaling pathway in the CSCs, resulting in the occurrence of plastic differentiation of CSCs	*in vitro* and *in vivo*	(33)
	ALDH^br^ cell population of Br-Ca-MZ01, MDA-MB-436, S68, SUM149, and SUM15	Salinomycin or salinomycin/JQ1 drug combination reduces the BCSC pool by inducing cell differentiation	*in vitro* and *in vivo*	(42)

**Table 2:** Anticancer mechanisms of salinomycin in BC cells.

Mechanisms	Cell lines	Molecular targets of salinomycin	Activity *in vitro/in vivo*	Ref.
Apoptosis	MCF7	Salinomycin inhibits the antiapoptotic genes BCL-2, BCL-XL, and BIRC5.	*in vitro*	(43)
	MCF7, T47D and MDA-MB-231	Salinomycin induces apoptosis through DNA damage, by which it activates *γ*H2AX, a marker of double strand breaks, and through downregulation of survivin expression.	*in vitro*	(44)
	MCF7, MDA-MB-231, Hs578T	Salinomycin increases DNA damage, by which the phosphorylated levels of p53 and *γ*H2AX was increased. Salinomycin also decreases the p21 protein level, which by the increased proteasome activity.	*in vitro*	(26)
	JIMT-1, MCF-7, and HCC1937	Salinomycin-induced ER Ca^2+^ depletion upregulates C/EBP homologous protein, which inhibits Wnt signaling by downregulating *β*-catenin. The increased cytosolic Ca2+ also activates protein kinase C, which has been shown to inhibit Wnt signaling.	*in vitro*	(37)
ROS induced autophagy and apoptosis	MCF-7, T47D, MDA-MB-453	Salinomycin leads to the formation of ROS eliciting JNK activation and induction of the transcription factor JUN. Moreover, salinomycin induces autophagy through the JNK pathway.	*in vitro*	(45)
	MCF-7, MDA-MB-231	Salinomycin-mediated ROS production leads to mitochondrial dysfunction and induces apoptosis and autophagy. Moreover, autophagy inhibition is involved in acceleration of apoptosis induced by salinomycin.	*in vitro*	(46)
Mitophagy and mitoptosis	SKBR3, MDAMB468	Salinomycin increases mitochondrial membrane potential (ΔΨ), decreases cellular ATP level, and induces mitophagy, mitoptosis	*in vitro*	(16)
Angiogenesis	MCF-7, T47D, MDA-MB-231, MDA-MB-468, 4T1	Salinomycin inhibits HIF-1*α* transcription factor activity and inhibits hypoxia-induced HIF-1*α*/VEGF signaling axis.	*in vitro* and *in vivo*	(47)
Cell proliferation	MCF-7, HS578T and MDA-MB-231 cells	Salinomycin inhibits LRP6, activates GSK3*β*, and suppresses the expression of cyclin D1 and survivin, two targets of both Wnt/*β*-catenin and mTORC1 signaling, displaying remarkable anticancer activity.	*in vitro*	(36)
	MCF7, T47D and MDA-MB-231	Salinomycin induces a p53-independent upregulation of p21^waf/cip^ and leads to growth arrest.	*in vitro*	(44)
	MCF7	Salinomycin inhibits cell proliferation *via* downregulation of Smo and Gli1 in Hedgehog–Gli1 signaling.	*in vitro*	(39)
	MDA-MB-231	Salinomycin inhibits cell proliferation by downregulating the expression of key elements Shh, Smo, and Gli1 in the Hedgehog pathway.	*in vitro*	(38)
	MCF7, T47D and MDA-MB-231	Slinomycin suppresses mammosphere formation and tumor growth *in vivo via* the inhibition of ALDH1 activity and downregulates the transcription factors Nanog, Oct4 and Sox2.	*in vitro* and *in vivo*	(34)

## Multiple Functions of Salinomycin in BC

Salinomycin and its derivatives have multiple functions in BC cells. We review evidence for its precise effects on various cellular processes in BC and the mechanisms underlying these effects.

Three major cell death pathways have been described: apoptosis, autophagy, and necrosis (48). Cancer cell death induced by salinomycin is achieved by multiple mechanisms (13, 16, 24). In fact, accumulating evidence shows that apoptosis and autophagic cell death can both occur, with crosstalk between the two pathways. Furthermore, both pathways may eventually lead to necrosis (secondary necrosis), depending on the stimulus and cell type (49).

### Effect of Salinomycin on the Apoptosis Pathway

Apoptosis is the major cell death pathway contributing to the removal of unnecessary and harmful cells during embryonic development, tissue homeostasis, and immune regulation. Apoptosis is mainly executed by intracellular cysteine proteases called caspases, which may be activated by two principal pathways: death receptor-dependent (extrinsic) and -independent (intrinsic or mitochondrial) pathways (50).

Salinomycin induces apoptosis in CSCs of different origins (18, 51, 52). However, the precise mechanisms vary substantially and are tightly correlated with the origin of the CSCs (26, 53–55).

Salinomycin increases DNA breaks in BC cells as well as the expression of phosphorylated p53 and γH2AX in Hs578T cells. It was indicated that salinomycin increases DNA damage, and this effect plays an important role in increasing apoptosis (26). Furthermore, salinomycin induces the expression of the pro-apoptotic protein Bax but inhibits the expression of the anti-apoptotic protein Bcl-2 in MDA-MB231 cells, suggesting that an increase in the Bax/Bcl-2 ratio may be involved in salinomycin-induced apoptosis (46). The same result was shown in cisplatin-resistant MCF7^DDP^ cells; in these cells, salinomycin abrogated nuclear translocation of NF-κB and caused a concurrent reduction in NF-κB-regulated expression of pro-survival proteins (27). Moreover, salinomycin reportedly activates a distinct apoptotic pathway that is not accompanied by cell cycle arrest and is independent of the tumor suppressor protein p53, caspase activation, the CD95/CD95L system, and proteasome (54). Interestingly, Yusra Al Dhaheri et al. reported that combination of salinomycin with paclitaxel or docetaxel can synergistically increase apoptosis in the BC cell line MDA-MB-231, which is not sensitive enough to salinomycin, providing a new insight for the clinical application of salinomycin (53).

These results suggest that the induction of apoptotic or non-apoptotic cell death by salinomycin in cancer cells depends on the particular cell type and that the detailed mechanisms underlying salinomycin-induced cell death in cancer cells remain to be fully elucidated.

### Effect of Salinomycin on the Autophagy Pathway

Autophagy is the catabolic process that regulates the degradation of a cell’s own components *via* the lysosomal machinery, characterized by the appearance of large autophagic vacuoles in the cytoplasm. It can be considered a physiological process used for the recycling of damaged organelles and energy supply during “lean times,” leading to cell death if extensively activated. At low levels, autophagy promotes cell survival by removing damaged proteins and organelles while supplying extra energy; however, the excessive and long-term upregulation of autophagy eventually results in the destruction of essential proteins and organelles beyond a certain threshold, resulting in cell death. Salinomycin reportedly induces and inhibits autophagy (56).

On the one hand, a substantial autophagic response to salinomycin (substantially stronger than the response to the commonly used autophagy inducer rapamycin) has been detected in BC cells (SKBR3 and MDA-MB-468 cells) and to a lesser degree in normal human dermal fibroblasts (16). Salinomycin strongly decreases ATP levels in BC cells in a time-dependent manner. Conversely, following treatment with salinomycin, human normal dermal fibroblasts exhibit decreased mitochondrial mass, although they are largely resistant to salinomycin-induced ATP depletion. Autophagy caused by salinomycin has a protective effect on various cancer cell lines, but experiments have shown that by blocking the expression of ATG7 with siRNA (41), this protective effect can be reversed. Therefore, the combined use of autophagy blockers in the clinic will be more effective.

On the other hand, the inhibitory effect of salinomycin on autophagy may explain its ability to block the degradation of LC3 and long-lived proteins. The suppressive effect of salinomycin on autophagic flux is relatively higher in the ALDH(+) population in HMLER cells than in the ALDH(−) population, and this differential effect is correlated with an increase in apoptosis in the ALDH(+) population. ATG7 depletion accelerates the proapoptotic capacity of salinomycin in the ALDH(+) population (41). Similarly, Pellegrini et al. found that CSC-like cells have greater sensitivity to autophagy inhibition than that of cells not expressing CSC markers (32).

### Effect of Salinomycin on Necrosis

Different from apoptosis, necrosis is another type of cell death that is typically not associated with the activation of caspases. It is characterized by the swelling of the endoplasmic reticulum (ER), mitochondria, and cytoplasm, with the subsequent rupture of the plasma membrane and cell lysis. Necrotic cell death is the result of interplay between several signaling cascades. The determinants of necrosis are mainly RIP3, calcium, and mitochondria. RIP3 interacts with RIP1 and binds to several enzymes involved in carbohydrate and glutamine metabolism. Calcium controls the activation of PLA, calpains, and NOS, inducing a series of events leading to necrotic cell death.

According to Xipell et al., in glioblastoma cells, salinomycin induces substantial ER stress, triggering the unfolded protein response and an aberrant autophagic flux that culminates in necrosis due to mitochondria and lysosomal alterations (57). However, the mechanism underlying necrosis in BC has not been revealed. It may occur as a consequence of massive cell injury or in response to extreme changes in physiological conditions.

### Effects of Salinomycin on Cell Cycle Arrest

Treatment with salinomycin hampers the proliferation of BC cells, and this effect is mediated by different mechanisms. For example, salinomycin inhibits proliferation in MDA-MB-231 cells in concentration- and time-dependent manners; in particular, it blocks the G1-to-S phase transition by the downregulation of genes downstream of the Hedgehog signaling pathway (38). In addition, salinomycin blocks the proliferation of BC cells by suppressing cyclin D1 expression and the GSK3*β*-mediated inhibition of the Wnt signaling pathway (36). It also suppresses BCSC proliferation, concomitant with the downregulation of cyclin D1 and increased p27(kip1) nuclear accumulation (34). Furthermore, an interesting study has shown that at low concentrations, salinomycin could induce transient G1 arrest at early time points and G2 arrest at late time points as well as senescence, in addition to inducing an enlarged cell morphology, the upregulation of p21 protein, and increases in histone H3 and H4 hyperacetylation and SA-*β*-Gal activity (44).

### Salinomycin and Reactive Oxygen Species (ROS)

ROS, which are products of normal metabolism and xenobiotic exposure, can be beneficial or harmful to cells and tissues depending on their concentration. ER stress can be an initiator of apoptosis and occurs when unfolded proteins accumulate; ER Golgi transport is inhibited, or the Ca^2+^ equilibrium is disrupted in cells. Excessive ROS production triggers oxidative modification of cellular macromolecules, inhibits protein function, and promotes cell death.

The elevated oxidative stress and mitochondrial membrane depolarization in response to salinomycin-mediated apoptosis were first reported in prostate cancer cells (55). Salinomycin can induce an increase in intracellular ROS levels, accompanied by decreased mitochondrial membrane potential, causing ER damage, with increased levels of Ca^2+^ being released into the cytoplasm; this process is regulated by Bcl-2 and Bax/Bak, further activating caspase-3 and the cleavage of PARP-1, ultimately leading to mitochondrial apoptosis. Moreover, Bcl-2, Bax, and Bak are also localized in the ER; thus, the ER serves as an important organelle for apoptotic control that is further enhanced by the mitochondria ER connection.

In BC cell lines (MCF-7, T47D, and MDA-MB-453), salinomycin induces ROS formation, causing JNK activation and induction of the transcription factor JUN. Salinomycin-mediated cell death was partially inhibited by *N*-acetyl-cysteine (NAC), a free radical scavenger, suggesting ROS formation contributes to the toxicity of salinomycin (45). This has been observed in MDA-MB-231 cells, in which salinomycin-mediated ROS production leads to mitochondrial dysfunction, and NAC attenuates salinomycin-induced apoptosis and autophagy. This result seems to conflict with the fact that the acceleration of apoptosis induced by salinomycin is related to autophagy inhibition. NAC thereby makes the function of salinomycin more complex. This indicates that the crosstalk between two different physiological responses (autophagy and apoptosis) induced by salinomycin might play a pivotal role in the determination of the fate of cancer cells (46).

Furthermore, ionomycin (AM5), a synthetic derivative of salinomycin, exhibits potent and selective activity against BCSCs *in vitro* and *in vivo* by accumulating and sequestering iron in lysosomes. Iron-mediated ROS production reportedly promotes lysosomal membrane permeabilization, thereby activating a cell death pathway consistent with ferroptosis (40). These findings highlight the importance of iron homeostasis in BCSCs, suggesting both iron- and iron-mediated processes to be potential therapeutic targets in BC.

### Effects on Tumor Cell Migration

Epithelial-to-mesenchymal transition (EMT) is the major cause of BC invasion and metastasis. In addition to substantial inhibitory effects on invasion and metastasis in BC, as determined by single-cell tracking, salinomycin significantly reduces the metastatic tumor burden in mice (58). Furthermore, various migration-related parameters in MDA-MB-231 cells are significantly lower after salinomycin treatment than in control cells, and the effects of salinomycin are concentration-dependent (59). Further, salinomycin inhibits the TGF-*β*1-induced EMT phenotypic transition and activation of Smad (p-Smad2/3 and Snail1) and non-Smad (*β*-catenin, p-p38, and MAPK) signaling molecules, which cooperatively regulate EMT induction. Importantly, these findings were confirmed in a series of BC specimens, in which there were strong correlations among levels of E-cadherin and *β*-catenin and the lymph node metastatic potential of BC (60).

### Effect on Neovascularization in BC

The characteristics of CSCs have been associated with angiogenesis. It has been reported that the expression of several CSC biomarkers correlates with that of angiogenesis markers, and some stem cell signaling pathways (*e.g.*, Notch) are used by both CSCs and by pro-angiogenic factors (61). This indicates that treatment linked to anti-angiogenic therapy and targeted at CSCs may be effective in the treatment of many malignant tumors, including BC. The antiangiogenic and anticancer efficacies of salinomycin in BC have been explored. In particular, salinomycin interrupts HIF-1*α*/VEGF signaling to inhibit VEGF-induced angiogenesis and BC growth. Moreover, it inhibits the expression of the pro-angiogenic cell surface marker CD31, thereby disrupting endothelial tubulogenesis, thereby interrupting endothelial tubulogenesis, decreases the binding of HIF-1*α* to the HRE sequence in human BC cells and suppresses neovascularization in a chick chorioallantoic membrane and a Matrigel plug-implanted mouse model (47). Salinomycin inhibits BC growth and tumor angiogenesis in mice based on bioluminescence and immunofluorescence imaging analyses. It also suppresses serum VEGFA levels in tumor-bearing mice and induces caspase-dependent apoptosis in BC cells (47).

### Effects on the Immune Microenvironment

Salinomycin could play a role in modulating the immune microenvironment in BC. It may exert these modulatory effects by two different mechanisms. First, at insufficient concentrations for direct antitumor activity, it could effectively stimulate M1 type macrophages and limit tumor growth and metastasis. In a previous study, the intratumoral injection of salinomycin increased the proportion of CD86 cells and decreased CD206 cells in transplant 4T1 tumors, thereby preventing tumor growth and pulmonary metastasis (62). Second, salinomycin could potently reverse tumor immune tolerance *via* the repression of IDO1 enzymatic activity. Moreover, it suppresses and inhibits IFN-*γ*-induced activation of the Janus kinase/signal transducer and activator of transcription (JAK/STAT) pathway and the nuclear factor NF-κB pathway, respectively, by inhibiting I*κ*B degradation and NF-κB phosphorylation. Furthermore, it restores the proliferation of T cells co-cultured with IFN-*γ*-treated BC cells and potentiates the antitumor activity of cisplatin *in vivo* (63).

## The Possible Molecular Mechanisms of Salinomycin

Salinomycin is a membrane ionophore that facilitates ion flux through the cytoplasmic and mitochondrial membranes. It works as a mobile carrier and discharges K^+^ rapidly (64–66). However, recent studies showed that salinomycin physically targets the lysosomal compartment, and not the ER, mitochondria, or the Golgi apparatus in CSCs (40, 67). It then accumulates in the lysosomal compartment in an endocytosis-independent manner (40). In the lysosomal compartment, salinomycin can interact with iron(II) and inhibit the effective translocation of the metal into the cytosol, thereby blocking the release of iron from lysosomes and resulting in lysosomal iron accumulation. The elevated iron level in lysosomes promotes enhanced ROS reaction *via* Fenton chemistry (68), possibly through lysosomal degradation of ferritin and the release of additional soluble redox-active iron, subsequently inducing lysosomal membrane permeabilization (LMP) (69). Compared with normal cells, cancer cells are sensitive to LMP through a variety of mechanisms, such as altered lysosomal localization, decreased LAMP-1 and LAMP-2 level (70), increased lysosomal size, altered heat shock protein 70 localization, and elevated sphingosine level (71–74). Particularly, CSCs contain significantly higher levels of iron, which either directly alter their differentiation or cause them to be selected for proliferation in favor of a subpopulation exhibiting a pronounced CSC phenotype.

**Figure 3:**
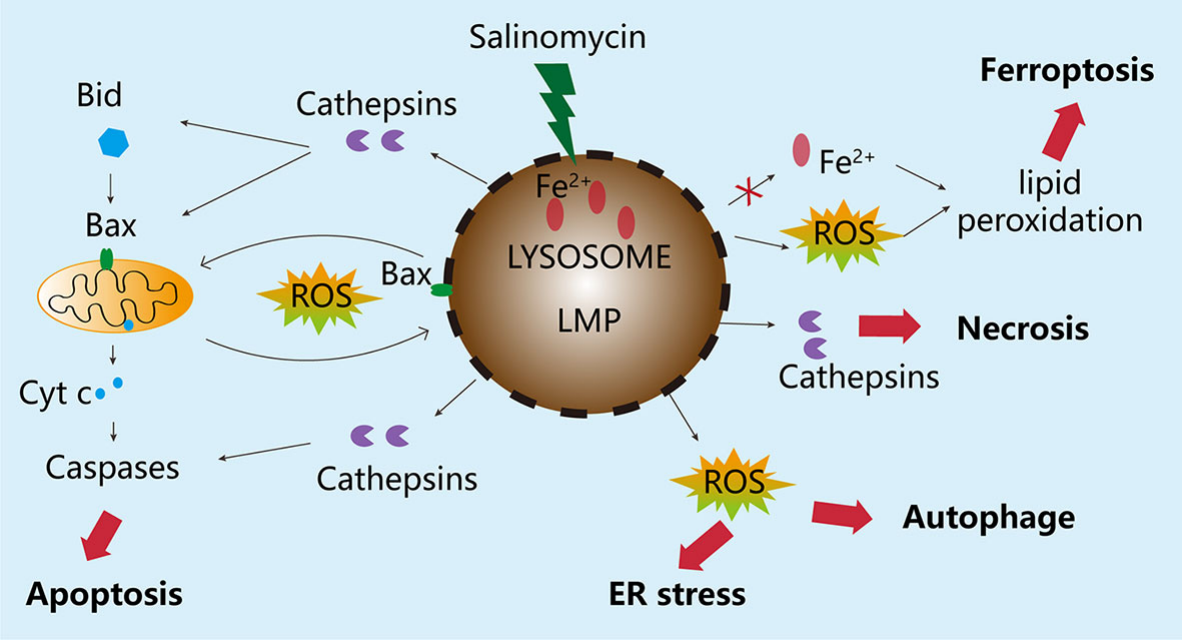
Molecular mechanisms underlying the effects of salinomycin through the lysosomal compartment. Salinomycin is enriched in the lysosome of breast cancer tumor cells, leading to imbalance in Fe+ metabolism, excessive accumulation of Fe ions, and degradation of ferritin, which causes the release of a large amount of free radicals and ultimately induces LMP. Depending on the degree of damage to the lysosome, different responses will be triggered. If the damage to the lysosome is minor, the stress sensor and response mechanisms, including autophagy, will be activated to ensure cell survival. However, when the degree of LMP is very high, the damage cannot be repaired. As a result, special cathepsins will be released from the lysosome into the cytoplasm, causing the disruption of mitochondrial membrane integrity, leading to apoptosis. In the case of severe damage to the lysosome, the release of enzymes from the lysosome can also cause uncontrolled cell death, such as necrosis edit from (69).

Therefore, in CSCs, salinomycin can trigger a much stronger ROS reaction and severe LMP. Salinomycin has been shown to activate distinct cell responses, including autophagy, apoptosis, and necrosis. This is likely linked to the extent of LMP in various cancer cells in response to salinomycin. Lysosomes are intrinsically heterogeneous, and not all lysosomes are permeabilized simultaneously in response to lysosomal stress (75, 76). Thus, damage to a small proportion of lysosomes can be fixed by activating lysophagy and other endolysosomal damage response mechanisms, thereby ensuring cell survival. However, when majority of lysosomes are damaged or autophagy is inhibited, the damaged lysosomes can no longer be eliminated by lysophagy, causing cell death. This point has not been addressed by previous investigations on salinomycin, and thus requires clarification. In some cancer cells, salinomycin triggered LMP and resulted in the translocation of cathepsins from lysosomes to the cytosol, where they induced the proteolytic activation of substrates, such as Bid and Bax, which in turn, promoted mitochondrial outer membrane permeabilization and caspase activation (77–80). This cell death can be inhibited by blocking cathepsin activity with protease inhibitors or by increasing the activity of iron chelators and endogenous cathepsin inhibitors, such as serpins and cystatins (81). However, under some circumstances, complete lysosomal rupture can also lead to uncontrolled necrosis. Ferroptosis is a regulated cell death pathway. A recent publication showed that salinomycin can kill CSCs by sequestering iron in lysosomes *via* a process triggered by severe lipid peroxidation related to ROS production and iron availability, and such cell death could be partially prevented by the ferroptosis inhibitor, ferrostatin-1, whereas the apoptosis and necrosis inhibitors Z-VAD-FMK and necrostatin-1, respectively, could not influence the cell death profiles (67). The possible molecular mechanisms underlying the effect of salinomycin through the lysosomal compartment on BC cells have been summarized in this review (see **Figure 3**).

## Application of Salinomycin in BC Therapy

As a potent therapeutic agent against cancer, salinomycin is still in the initial phase of the preclinical stage. Up to date, there is still no registered clinical trial about salinomycin. Two individual cases have been described in the literature as follows: one describing a 40-year-old female patient with metastatic (bone and subcutaneous) invasive ductal BC and another describing an 82-year-old female patient with advanced and metastatic (pelvic lymphatic metastasis) squamous cell carcinoma of the vulva. Both cases were treated systemically with intravenous administration of salinomycin (200–250 μg/kg salinomycin every second day), which partially regressed metastasis for 3 weeks and showed only minor adverse effects, as opposed to the severe adverse effects commonly observed with conventional chemotherapy (24). These findings confirm the safety and efficacy of salinomycin for selective clinical use. However, the potential toxicity of salinomycin may need to be studied in larger clinical trials.

## Potential Combination Therapy With Salinomycin

Tumors are composed of heterogenous cell populations with various mutations and/or phenotypes. Therefore, it is generally ineffective to use a single drug to prevent cancer progression. For the complete eradication of cancers, several drugs with distinct mechanisms but complementary anticancer activities (*i.e.*, combination therapies) are often used to enhance the antitumor efficacy and minimize the risk of developing drug resistance. The recent development of innovative delivery methods using biocompatible nanocarriers has enabled studies of various combinations. In particular, combination therapy with salinomycin can achieve promising results, and selective delivery *via* biocompatible nanocarriers provides a novel therapeutic approach (82–85). The effects of various combination strategies with salinomycin on BC cells have been summarized in **Table 3**.

**Table 3:** Synergistic anticancer co-action of salinomycin with other agents in BC.

Synergy	Cell lines	Effect against cancer cells	Activity *in vitro*/*in vivo*	Ref.
Conventional chemotherapy	Hs578T	Salinomycin sensitizes paclitaxcel-, docetaxcel-, vinblastin-, or colchicine-treated cancer cell lines; on one side salinomycin increases pH2AX level and reduces p21 level; this leads to mitotic catastrophe; on the other side, salinomycin reduces cyclin D1 level to prevent G2 arrest.	*in vitro*	(53)
	MDA-MB-231 or MCF7 mammospheres	Combination therapies of salinomycin with paclitaxel or lipodox show a potential to improve tumor cell killing	*in vitro*	(86)
	MDA-MB-231, MCF7 and Hs578T	Salinomycin increases the sensitivity of cancer cells to the apoptotic effects of doxorubicin or etoposide	*in vitro*	(26)
	MCF7 cells and MCF7-MS	Nanoparticles loaded with salinomycin and docetaxel at molar ratio of 1:1 is synergistic.	*in vitro* and *in vivo*	(87)
	MCF7 side population cells	Octreotide-modified paclitaxel-loaded PEG-b-PCL polymeric micelles and salinomycin-loaded PEG-b-PCL polymeric micelles combination has the strongest antitumor efficacy.	*in vitro* and *in vivo*	(88)
	MDA-MB-231, MCF7	Combination of hyaluronic acid-coated salinomycin nanoparticles and paclitaxel nanoparticles shows the highest cytotoxicity against CD44(+) cells	*in vitro*	(89)
	MDA-MB-231 CD44^+^/CD24^-/low^ CSCs and no CSCs	Single-walled carbon nanotubes conjugated to CD44 antibodies with salinomycin and paclitaxel could target and eradicate both whole tumor cells and CSC populations.	*in vitro* and *in vivo*	(85)
	MCF7	Hyaluronic acid-coated vitamin E-based redox-sensitive salinomycin prodrug nanoparticles were fabricated to deliver paclitaxel for cancer-targeted and combined chemotherapy, and this led to maximize the chemotherapeutic effect	*in vitro*	(90)
HER2	BT-474 and MDA-MB-361 and breast cancer xenografts	Sali-NP-HER2 nanoparticles efficiently bound to HER2-positive BCSCs and BC cells, resulting in enhanced cytotoxic effects compared with non-targeted nanoparticles or salinomycin	*in vitro* and *in vivo*	(91)
	MCF7	Herceptin-immobilized salinomycin-encapsulated poly (lactic-co-glycolic acid) nanoparticles could successfully be uptaken by MCF7 cells	*in vitro*	(92)
	MCF7 mammospheres	Combinatorial treatment of mammospheres with trastuzumab and salinomycin is superior to single treatment with each drug	*in vitro*	(93)
Estrogen receptor	MCF7, T47D	Salinomycin induces an additional cytotoxic effect when treated combinational with tamoxifen by inhibiting the ligand independent activation of Er*α*.	*in vitro*	(94)
	MCF7/LCC2 and MCF7/LCC9 cell lines	Salinomycin has synergistic effect with tamoxifen and enhanced tamoxifen sensitivity by decreasing AIB1 expression	*in vitro*	(95)
Small molecule inhibitor	MDA-MB-468, MDA-MB-231, MCF7	The combination of salinomycin and dasatinib (a Src kinase inhibitor) shows enhanced potency against human BC cell lines and tumor spheroids.	*in vitro*	(96)
	HCC1937 cells	Combination of LBH589(a histone deacetylase inhibitor) and salinomycin has a synergistic inhibitory effect on TNBC BCSCs by inducing apoptosis, arresting the cell cycle, and regulating EMT.	*in vitro* and *in vivo*	(82)
	Hs578T	Co-treatment of salinomycin sensitizes AZD5363 (an inhibitor of protein kinase B)-treated cancer cells through increased apoptosis	*in vitro*	(97)
Phytoalexin	MDA-MB-231, MDA-MB-468 and T47D	Resveratrol combination with salinomycin exerts synergistic anti-proliferative activity on BC cells.	*in vitro*	(98)
	MDA-MB-231	Resveratrol and salinomycin inhibit EMT (fibronectin, vimentin, N-cadherin, and slug); chronic inflammation (Cox2, NF-kB, p53), autophagy (Beclin and LC3) and apoptotis (Bax, Bcl-2) markers.	*in vitro* and *in vivo*	(99)

Salinomycin and dasatinib (a Src kinase inhibitor) have a synergistic effect in BC cells *via* the horizontal suppression of multiple pathways (96). This effect possibly involves the promotion of cell cycle arrest at the G1/S phase through both the estrogen-mediated S phase entry and BRCA1 and DNA damage response pathways.

Additionally, salinomycin increases cancer cell sensitivity to the apoptotic effects of doxorubicin (DOX) or etoposide (ETO). pH2AX, pBRCA1, p53BP1, and pChk1 levels substantially increase in response to co-treatment with salinomycin and either DOX or ETO. The level of the anti-apoptotic p21 protein increases in response to DOX or ETO but decreases in response to salinomycin, which increases proteasome activity. These results indicate that the ability of salinomycin to sensitize cancer cells to DOX or ETO is associated with an increase in DNA damage and a decrease in p21 levels. Accordingly, salinomycin-based chemotherapy may be beneficial for patients with cancer receiving DOX or ETO (26).

The development of innovative delivery systems has significantly improved the efficacy of combination therapy (100). For example, Gao et al. (87) found that the synergistic effects of salinomycin and docetaxel could be effectively maintained *in vivo* by the co-encapsulation of these agents in PLGA/TPGS nanoparticles, providing a promising strategy for BC treatment.

Moreover, combining therapies targeting CSCs with conventional chemotherapy may be a useful strategy for cancer treatment (83). A single-walled carbon nanotube (SWCNT) drug delivery system combining paclitaxel, salinomycin, and biocompatible CD44 antibody-conjugated SWCNTs *via* a hydrazone linker had better effects than did individual drug-conjugated nanocarriers or free drug suspensions in both *in vitro* and *in vivo* assays (85). Moreover, another study has shown that an elastin-like polypeptide-salinomycin nanoparticle has a long release half-life *in vitro* and results in a lower CSC frequency in 4T1 orthotopic tumors than in untreated tumors or free salinomycin-treated tumors (84). Furthermore, HER2 is overexpressed in both breast CSCs and cancer cells and can also be utilized for targeted delivery systems. Salinomycin-loaded polymer-lipid hybrid anti-HER2 nanoparticles (Sali-NP-HER2) were developed to target both HER2-positive breast CSCs and cancer cells, with a high delivery efficacy and low untargeted toxicity (91).

Simultaneous eradication of both CSCs and cancer cells is necessary for optimizing therapeutic efficacy (101). The combination of salinomycin and traditional therapies has proven to be effective in BC; however, the proximal mechanisms underlying the effects of salinomycin on CSCs remain unclear and should be a focus of future studies.

## Conclusion

The potency of tumor initiation and drug resistance of BCSCs are key factors contributing to the metastasis and recurrence of BC and thus increasing mortality. Salinomycin in combination with traditional chemotherapy and targeted therapy can kill both common cancer cells and CSCs, providing a very promising clinical strategy to treat BC. Salinomycin has multiple functions in the regulation of cellular processes in BC cells. Importantly, it functions in the regulation of cell death by a highly complex mechanism involving multiple pathways and interactions among several cell death-related biological processes. Its effects may be mediated by Bax/Bak, the activation of mitoptosis, irreversible deterioration of mitochondrial structure, or other apoptogenic factors associated with apoptosis released from mitochondria. Owing to the limited research focused on the mechanisms of action of salinomycin, further studies of its precise effects on apoptosis and autophagy, as well as the interactions among mitoptosis, mitophagy, ferroptosis, and necrosis are needed. In addition, suitable methods for the identification and isolation of CSCs are lacking despite their key importance for studies of the effects of salinomycin and the development of CSC-targeted therapy. Furthermore, a better understanding of the different characteristics of somatic stem cells and CSCs, such as the signaling pathways that regulate self-renewal and cell fate, would be important for the design of new strategies targeting CSCs.

## Author Contributions

HW, HZ, and FC: conceptualization. HW, HZ, and CC wrote the manuscript. YZ, ZW, and FC: manuscript review and critical comments. All authors contributed to the article and approved the submitted version.

## Funding

This review article was supported by the National Natural Science Foundation of China (no. 81802961), the Shanghai Yangpu District Health and Family Planning Commission Fund for Hao Yi Shi Training Project (2020-2023), and the Natural Science Foundation of Shanghai (grant no. 18ZR1436000).

## Conflict of Interest

The authors declare that the research was conducted in the absence of any commercial or financial relationships that could be construed as a potential conflict of interest.

## Publisher’s Note

All claims expressed in this article are solely those of the authors and do not necessarily represent those of their affiliated organizations, or those of the publisher, the editors and the reviewers. Any product that may be evaluated in this article, or claim that may be made by its manufacturer, is not guaranteed or endorsed by the publisher.

## Glossary

**Table:**

AIB1	amplified in breast cancer 1
ALDH	acetaldehyde dehydrogenase
ATG7	autophagy related protein 7
ATP	adenosine triphosphate
BCSCs	breast cancer stem cells
BIRC5	baculoviral IAP repeat containing 5
C/EBP	CCAAT/enhancer binding protein
CHOP	C/EBP homologous protein
Cox2	cyclooxygenase-2
CSCs	cancer stem cells
EMT	epithelial-mesenchymal transition
ER	endoplasmic reticulum
Er*α*	estrogen receptor *α*
GSK3*β*	glycogen synthase kinase-3 *β*
H2AX	H2A histone family member X
HIF-1	hypoxia inducible factor 1
JNK	Jun N-terminal kinase
LC3	the microtubule-associated protein 1 light chain 3
LMP	lysosomal membrane permeabilization
LRP6	LDL-receptor-related protein 6
mTORC1	mechanistic target of rapamycin complex 1
NF-kB	nuclear factor κ-light chain-enhancer of activated B cells
PARP1	poly(ADP-ribose) polymerase 1
PEG-b-PCL	poly (ethylene glycol)-block-poly (*ε*-caprolactone)
PKCα	protein kinase C *α*
PTCH	patched homolog
ROS	reactive oxygen species
Sali-NP-HER2	salinomycin-loaded polymer-lipid hybrid anti-HER2 nanoparticles
SMO	smoothened
STAT3	signal transducer and activator of transcription 3
TNBC	triple-negative breast cancer
VEGF	vascular endothelial growth factor
Wnt	wingless/integrated
